# Tyrosine modification increases the affinity of gastrin for ferric ions

**DOI:** 10.1186/s40064-015-1622-2

**Published:** 2015-12-24

**Authors:** Graham S. Baldwin, Ioulia Sims

**Affiliations:** Department of Surgery, Austin Health, The University of Melbourne, Heidelberg, VIC 3084 Australia

**Keywords:** Gastrin, Ferric, Iron, Phosphorylation, Sulphation

## Abstract

The peptide hormone gastrin_17_, which occurs naturally in both tyrosine sulphated and unsulphated forms, binds two ferric ions with pM affinities. The aim of this study was to investigate the hypothesis that sulphation or phosphorylation of gastrin_17_ altered ferric ion binding, and/or affinity for the CCK1 or CCK2 receptor. To investigate the effect of tyrosine modification on ferric ion binding, the changes in absorbance of gastrin_17_, gastrin_17_SO_4_ and gastrin_17_PO_4_ on addition of Fe^3+^ ions were monitored. Binding of gastrin_17_, gastrin_17_SO_4_ and gastrin_17_PO_4_ to the human CCK1 and CCK2 receptors was assessed by competition with [^125^I]-Bolton and Hunter-labelled cholecystokinin_8_ in transiently transfected COS cells. Tyrosine sulphation or phosphorylation increased the affinity of gastrin_17_ for the first ferric ion bound from 267 to 83 pM and 14 pM, respectively, but had no effect on the stoichiometry of ferric ion binding. In contrast the affinity of gastrin_17_ for the second ferric ion bound was reduced from 94 pM to 7.32 µM and 671 nM, respectively. While sulphation of gastrin_17_ increased its affinity for the CCK2 receptor approximately 50 fold, phosphorylation had no effect on receptor binding. These results demonstrate that tyrosine modification may have profound effects on the interaction of gastrins with ferric ions and with the CCK2 receptor.

## Background

The classical gastrointestinal hormone gastrin_17_ (ZGPWLEEEEEAYGWMDFamide, Gamide) was first recognized by its ability to stimulate gastric acid secretion (Dockray et al. 2001). Human gastrin is initially synthesized as a 101 amino acid preprohormone, which is processed via the 80 amino acid prohormone progastrin, to various non-amidated precursors including glycine-extended gastrin_17_ (ZGPWLEEEEEAYGWMDFG, Ggly) (Dockray et al. 2001). While Gamide stimulates proliferation in the gastric mucosa (Koh et al. 1999), progastrin and Ggly are important growth factors for the colorectal mucosa (Aly et al. 2004). The related hormone cholecystokinin (CCK), which has the same amidated C-terminal pentapeptide sequence as gastrin_17_, is responsible for gallbladder contraction and pancreatic enzyme secretion (Miyasaka and Funakoshi 2003).

The receptors for CCK (CCK1 receptor) and Gamide (CCK2 receptor) are both members of the seven transmembrane domain family, and share 50 % sequence identity (Aly et al. 2004; Shulkes and Baldwin 1997). The CCK1 receptor is found on pancreatic acini and has a 1000-fold higher affinity for sulphated CCK_8_ (CCK_8_SO_4_) than for unsulphated CCK_8_. However, sulphation of CCK_8_ does not greatly affect its affinity for the CCK2 receptor, which is found in the gastric mucosa and in the brain (Shulkes and Baldwin 1997). Neither the CCK1 nor the CCK2 receptor recognizes non-amidated forms of CCK or gastrin, but the receptors can be readily distinguished with several selective antagonists (Shulkes and Baldwin 1997).

Gastrins such as Ggly and Gamide bind two ferric ions (Baldwin et al. 2001), the first to Glu7 and the second to Glu8 and Glu9 (Baldwin et al. 2015; Pannequin et al. 2002). Ferric ions are essential for the biological activity of Ggly as a stimulant of cell proliferation and migration (Pannequin et al. 2002), but are not required for the biological activity of Gamide (Pannequin et al. 2004). For example, treatment with the iron chelator desferrioxamine, or substitution of Glu7 with Ala, completely blocked the biological activity of Ggly (Pannequin et al. 2002), but had no effect on the biological activity of Gamide (Pannequin et al. 2004).

Sulphation of CCK_8_ on the sole tyrosine residue greatly increases receptor binding and biological potency (Jensen et al. 1981). Tyrosine sulphation or phosphorylation of CCK_8_ increased the stoichiometry of ferric ion binding from 1 to 2, without greatly affecting the affinity (Baldwin et al. 2008). Gastrins also occur in sulphated and unsulphated forms (Dockray et al. 2001), and the sulphated form predominates in the fetal pancreas (Brand et al. 1984). Gastrin can also be phosphorylated by the EGF receptor tyrosine kinase in vitro (Baldwin et al. 1983), but phosphorylated gastrins have not been reported to occur naturally. The aim of the present study was to determine whether or not sulphation or phosphorylation of gastrin_17_ altered ferric ion binding or affinity for the CCK1 or CCK2 receptor. Since non-amidated forms of gastrin require ferric ions for activity, a change in affinity for ferric ions on modification of the tyrosine residue of gastrins might have profound effects on their biological activity.

## Methods

### Peptides

Gastrin_17_ and sulphated gastrin_17_ (95 and 96 % pure, respectively) were purchased from Bachem (Bubendorf, Switzerland). Phosphorylated gastrin_17_ (85 % pure) was from Mimotopes (Clayton, Australia). All peptides had a pyroglutamyl residue at their N-terminus and were C-terminally amidated. The impurities consisted of water and salts.

### Absorption spectroscopy

The absorption of peptides (10 μM in 10 mM sodium acetate (pH 4.0) containing 100 mM NaCl and 0.005 % Tween 20) at 280 nm in the presence of increasing concentrations of ferric ions from 2.5 to 50 μM was measured against a buffer blank, in 1 ml quartz cuvettes thermostatted at 298 K, with a Cary 5 spectrophotometer (Varian, Mulgrave, Australia).

### Receptor binding assay

Binding of ligands to the human CCK1 or CCK2 receptors on transiently transfected COS-7 cells was measured by competition with sulfated [^125^I]-Bolton and Hunter labelled-CCK_8_ (50,000 cpm/well, Amersham Biosciences, Castle Hill, Australia) as described previously (Baldwin et al. 2008).

### Curve fitting and statistics

Data [expressed as mean ± standard error of the means (SEM)] for the binding of ferric ions to gastrins were fitted to a two-site ordered model with the program BioEqs (Royer 1993; Royer et al. 1990). Receptor binding data were analyzed by one-way analysis of variance, followed by Bonferroni’s *t* test. Differences with *P* values <0.05 were considered significant.

## Results and discussion

### Changes in absorbance on binding of ferric ions to tyrosine-modified gastrin_17_

**Fig. 1:**
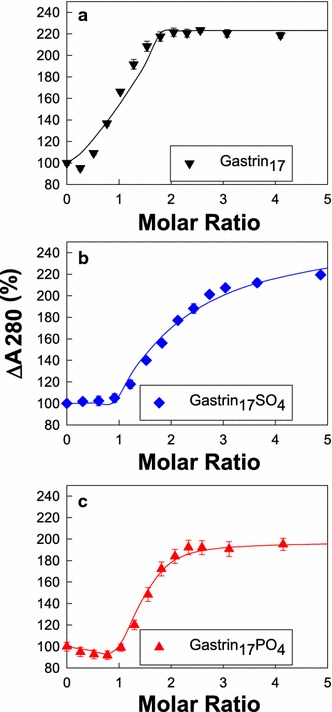
Tyrosine sulphation of gastrin enhances ferric ion binding. As reported previously, addition of aliquots of ferric chloride to 10 µM gastrin_17_ (**a**, *down triangle*) in 10 mM sodium acetate, 100 mM NaCl, 0.005 % Tween 20, pH 4.0 at 298 K resulted in an increase in the absorption at 280 nm up to a molar ratio of 2. Addition of aliquots of FeCl_3_ to 10 µM gastrin_17_SO_4_ (**b**, *diamond*), or gastrin_17_PO_4_ (**c**, *up* *triangle*) caused a more gradual change in absorption. Data are expressed as a percentage of the absorbance of that peptide without ferric ions. Points are means of at least three separate experiments; *bars* represent the SEM. *Lines* represent the best fit to a two site model with the program BioEqs; the appropriate K_d_ values are given in Table 1

The effect of addition of Fe^3+^ ions on the absorption spectrum of gastrin_17_, gastrin_17_SO_4_ and gastrin_17_PO_4_ was first investigated by absorption spectroscopy. A pH value of 4.0 was chosen in order to avoid any problems with precipitation of ferric hydroxides, and to allow direct comparison with previous studies. As reported previously (Baldwin et al. 2001, 2015) the absorption of gastrin_17_ at 280 nm increased to a maximum of 220 % after the addition of 2.0 mol ferric chloride/mol peptide (Fig. 1a). Fitting of the absorption data to a two-site ordered model with the program BioEqs gave values for K_d_1 and K_d_2 of 267 and 94 pM, respectively, in good agreement with the values determined previously (Baldwin et al. 2015) (Table 1). The increase in absorption of gastrin_17_SO_4_ (Fig. 1b) on addition of ferric ions was more gradual than for gastrin_17_, and in the case of gastrin_17_PO_4_ a slight decrease in absorption was initially observed (Fig. 1c). Nevertheless the absorption for both gastrin_17_SO_4_ and gastrin_17_PO_4_ finally reached maxima of 195 % to 220 % after the addition of 2–3 mol ferric chloride/mol peptide. Fitting of the absorption data to a two-site ordered model with the program BioEqs gave values for K_d_1 and K_d_2 of 83 pM and 7.32 µM, respectively, for gastrin_17_SO_4_, and of 14 pM and 671 nM, respectively, for gastrin_17_PO_4_ (Table 1).

**Table 1:** Binding of ferric ions by gastrin_17_, CCK_8_ and their derivatives

	Reference	Absorption			
		K_d_1 (pM)	K_d_2 (pM)	A_280_ Site 1 (%)	A_280_ Site 2 (%)
Gastrin_17_		267	94	100	223
Gastrin_17_	Baldwin et al. (2015)	300	85	100	313
Gastrin_17_SO_4_		83	7,320,000	88	259
Gastrin_17_PO_4_		14	671,000	98	215
CCK_8_	Baldwin et al. (2008)	Not detected			
CCK_8_SO_4_	Baldwin et al. (2008)	Not detected			
CCK_8_PO_4_	Baldwin et al. (2008)	Cooperative	Cooperative		194^a^

The affinity of, and the percentage absorbance change at 280 nm on, the binding of the first and second ferric ions to gastrin_17_, gastrin_17_SO_4_ or gastrin_17_PO_4_ at pH 4.0 were determined by fitting the mean data obtained in the absorbance experiments (N = 3) described in the Fig. 1 legend to a two site ordered model with the program BioEqs

^a^The absorbance changes for CCK_8_ and its derivatives were measured at 275 nM as the peptide contains tyrosine and phenylalanine, but no tryptophan, residues (Baldwin et al. 2008)

This data indicates that, although tyrosine sulphation or phosphorylation had no effect on the stoichiometry of ferric ion binding, the affinity of gastrin for the first ferric ion was enhanced, and for the second ferric ion was reduced, by modification of the tyrosine side chain. The possibility that the hydroxyl group of the tyrosine is itself a ligand for the second ferric ion does not appear to be likely as XAFS data suggested that three of the five glutamate side chains act as ferric ion ligands (Baldwin et al. 2015). A more likely explanation is that the bulky sulphate and phosphate groups occupy the ferric ion binding site and prevent access of more than one ferric ion.

The concentrations of gastrins and of ferric ions used in the above experiments were supra-physiological. The concentration of gastrins was chosen as the minimum concentration that gave a reproducible change in absorbance on addition of ferric ions. Nevertheless our previous publications indicate that the binding of ferric ions to gastrin at *physiological* concentrations is essential for biological function. Thus mutation of the glutamate residues essential for binding ferric ions abrogates the biological activity of Ggly as a stimulant of cell proliferation and migration (Pannequin et al. 2002). Similarly removal of ferric ions with the iron chelator desferrioxamine (Ferrand et al. 2010; Pannequin et al. 2002), or by competition with bismuth ions (Kovac et al. 2012; Pannequin et al. 2002), blocked the biological activity of Ggly in vitro and in vivo.

### Effect of tyrosine modification on CCK receptor binding

**Fig. 2:**
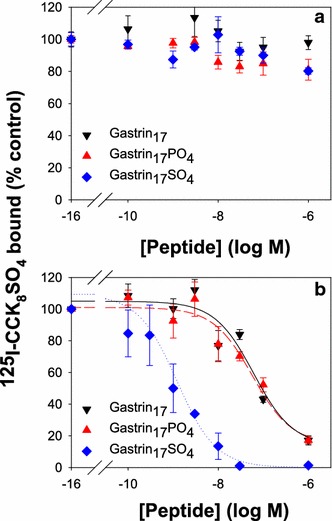
Tyrosine sulphation of gastrin enhances CCK2 receptor binding. The ability of gastrin_17_ (*down triangle*), gastrin_17_SO_4_ (*diamond*), or gastrin_17_PO_4_ (*up triangle*), to compete with [^125^I]-Bolton and Hunter labelled-CCK_8_SO_4_ (150 pM, 100,000 cpm) for binding to the human CCK1 (**a**) or CCK2 (**b**) receptor on transiently transfected COS-7 cells was measured as described in “Methods”. Points represent the mean data from at least three experiments, each in triplicate, and lines represent the best fit to a one site model. None of the three peptides competed with [^125^I]-CCK_8_SO_4_ for binding to the CCK1 receptor. The IC_50_ values for the binding of gastrin_17_, gastrin_17_SO_4_ and gastrin_17_PO_4_ to the CCK2 receptor were 61 ± 32, 1.2 ± 0.4 and 58 ± 20 nM, respectively. In contrast to the previously reported enhancement of binding to both receptors on sulphation of CCK_8_, phosphorylation had no effect on the peptide’s affinity for the CCK2 receptor

The effect of tyrosine modification on the binding of gastrin_17_ to either human CCK1 or CCK2 receptors was then examined. Although sulphation of gastrin_17_ increased its affinity for the human CCK2 receptor, phosphorylation of gastrin_17_ had no effect on CCK2 receptor binding (Fig. 2b). The IC_50_ values for the binding of gastrin_17_, gastrin_17_SO_4_ and gastrin_17_PO_4_ to the CCK2 receptor were 61 ± 32, 1.2 ± 0.4 and 58 ± 20 nM, respectively. The IC_50_ values for the binding of gastrin_17_ were slightly higher than the values, which range from 0.94 to 6.4 nM, previously reported for the cloned human CCK2 receptor (Ito et al. 1993; Lee et al. 1993; Pisegna et al. 1992). The affinity of gastrin_17_SO_4_ for the cloned human CCK2 receptor does not appear to have been reported previously, but data for binding to dispersed gastric glands from the guinea pig fundus also suggest that sulphation increases the affinity for the CCK2 receptor, in that case by a factor of tenfold, from 16 to 1.6 nM (Praissman et al. 1983). Binding of gastrin_17_, gastrin_17_SO_4_ and gastrin_17_PO_4_ to the CCK1 receptor was not detected in our experiments (Fig. 2a). In the case of gastrin_17_ this result agrees with the data of de Weerth and co-workers, who reported an IC_50_ value of 1.8 µM (de Weerth et al. 1993); binding affinities of gastrin_17_SO_4_ and gastrin_17_PO_4_ for the CCK1 receptor do not appear to have been reported previously.

### Comparison with CCK_8_

In contrast to CCK_8_, where tyrosine sulphation or phosphorylation increased the stoichiometry of ferric ion binding from 1 to 2 without greatly affecting the affinity (Baldwin et al. 2008), tyrosine sulphation or phosphorylation of gastrin_17_ had no effect on the stoichiometry of ferric ion binding, but enhanced the affinity of gastrin_17_ for the first ferric ion, and reduced the affinity for the second ferric ion. This difference is not unexpected as the gastrin binding site consists of three of the five glutamate residues, while the CCK_8_SO_4_ binding site involves the two aspartate residues, and the CCK_8_PO_4_ binding site the phosphate group itself (Baldwin et al. 2008). In terms of receptor binding, tyrosine sulphation enhanced the affinity, while phosphorylation reduced the affinity, of CCK_8_ for both the CCK1 and CCK2 receptors (Baldwin et al. 2008). In contrast, while tyrosine sulphation enhanced the affinity of gastrin_17_ for the CCK2 receptor, phosphorylation had no effect. Although the generalization that modification of tyrosine profoundly affects the binding of ferric ions is valid, the differences between two such closely related peptides as gastrin_17_ and CCK_8_ indicate that the exact binding details will have to be determined for each peptide individually.

## Conclusions

While tyrosine sulphation or phosphorylation of gastrin_17_ had no effect on the stoichiometry of Fe^3+^ ion binding, the affinity for the first ferric ion bound was increased from 267 to 83 pM and 14 pM, respectively. Sulphation of gastrin_17_ increased its affinity for the CCK2 receptor approximately 50 fold, but phosphorylation had no effect on receptor binding. These results imply that tyrosine modification may have profound effects on the biological activities of gastrins.

## Abbreviations

CCK: cholecystokinin
DMEM: Dulbecco’s Modified Eagle Medium
Gamide: amidated gastrin_17_
Ggly: glycine-extended gastrin_17_
